# Markers of immunosenescence in CMV seropositive healthy elderly adults

**DOI:** 10.3389/fragi.2024.1436346

**Published:** 2025-01-23

**Authors:** Ivón Johanna Rodríguez, Carlos Alberto Parra-López

**Affiliations:** ^1^ Grupo de profundización en Kinesioterapia, Departamento de Movimiento Corporal Humano, Universidad Nacional de Colombia, Bogotá, Colombia; ^2^ Grupo de Inmunología y Medicina Traslacional, Departamento de Microbiología, Universidad Nacional de Colombia, Bogotá, Colombia

**Keywords:** immunosenescence, T cells, NK cells, monocytes, flow cytometry

## Abstract

A significant increase in life expectancy has accompanied the growth of the world’s population. Approximately 10% of the global population are adults over 60, and it is estimated that 2050 this figure will double. This increase in the proportion of older adults leads to a more significant burden of age-related diseases. Immunosenescence predisposes elderly individuals to a higher incidence of infectious and chronic non-communicable diseases with higher mortality rates. Despite advances in research, it is necessary to evaluate the cellular characteristics of the aging immune system in populations with a high incidence of latent viruses such as cytomegalovirus (CMV). In this sense, this work aimed to identify senescence markers in cells of the innate and adaptive immune system in healthy older adults with CMV infection. We observed that older adults present an increase in the population of CD14^+^CD16^+^ intermediate monocytes, an expansion of CD56neg NK cells with an increase in the expression of CD57, as well as a decrease in the naïve CD4^+^ and CD8^+^ T cells, accompanied by an increased expression of senescence markers CD57 and KLRG1 in effector CD8^+^ T cells.

## 1 Introduction

Aging represents a paradoxical state of immunodeficiency and inflammation, which increases susceptibility to infections and chronic diseases, a reduced response to vaccination, and an increased risk of cancer (Nikolich-Zugich, 2018). These age-related changes in the immune system, which result in poor surveillance of tumors, infectious agents, and increased susceptibility to autoimmunity, are known as immunosenescence (Pereira et al., 2020). Immunosenescence is a multifactorial process that depends on environmental factors, exposure to antigens, and epigenetic changes associated with the unique immunological experience of everyone (immunobiography) (Franceschi et al., 2017). In this process, the accumulation of effector and memory cells in lymphoid organs, due to continuous stimulation and exposure to antigens, is characterized by several alterations, including decreased responsiveness to new antigens, non-sustained memory responses, expansion of terminally differentiated cells and chronic low-grade inflammation (Ventura et al., 2017; Goronzy and Weyand, 2013). Heterogeneity in immune system responses increases due to continuous remodeling of the immune system throughout life, contributing to the increased risk of infections, cancer, and autoimmune diseases in aging. These age-related changes affect both innate and adaptive immunity and generate deterioration in the functions and activities of the immune system (Franceschi et al., 2018).

Since 2000, the WHO has proposed health policies to prevent or compensate for immunological defects as an objective of healthy aging (OMS. Envejecimiento y salud, 2018). In this sense, research on immune aging seeks to find characteristics of senescence that can be manipulated or reversed to improve the quality of life of older adults. Infection by latent viruses such as cytomegalovirus has been described as a factor to consider when studying aging (Rodríguez et al., 2020). In Colombia, the incidence of cytomegalovirus is high. Therefore, we wanted to evaluate the characteristics of immunosenescence in a cohort of healthy CMV+ adults.

## 2 Materials and methods

### 2.1 Blood samples

The study was approved by the ethics committee of the Facultad de Medicina_Universidad Nacional de Colombia–Bogotá (reference number ACT-011-164-18 of 15 Jun 2018). We invited 20 healthy adults. The following inclusion criteria were defined: healthy volunteers aged 18–30 years and healthy adults over 60 residing in Bogotá, Colombians. Non-inclusion criteria: Recent acute infection, consumption of antibiotics 3 weeks prior to sample collection, consumption of anti-inflammatory medications and corticosteroids, autoimmune diseases, immunodeficiencies, cancer, Diabetes mellitus, cirrhosis, alcoholism, and smoking. Participants refrained from strenuous physical activity 24 h before sampling. A group of 10 young people (18–28 years old with an average age of 24.5 ± 2.98 years, five men and five women) and a group of ten older adults (60–85 years old with a mean age of 67.9 ± 9.07 years, five men and five women) Table 1. All participants lived independently in the community, without health problems or disabilities, and gave written informed consent before study entry. Afterward, peripheral venous blood (60 mL) was obtained in heparinized tubes. PBMCs were isolated using a density gradient (Histopaque, Sigma Aldrich) and were cryopreserved in AIM-V 50% (Gibco, ThermoFisher), FBS 40% (Gibco, ThermoFisher), and DMSO 10% (MP Biomedicals, LLC) in liquid nitrogen until use.

**TABLE 1:** Key demographic variables of donors included in the study.

Donor ID	Age (yr)	Sex	Status CMV
JO1	25	F	Positive
JO2	23	F	Positive
JO3	18	M	Positive
JO4	25	F	Positive
JO5	21	M	Positive
JO6	24	F	Positive
JO7	28	M	Positive
JO8	24	F	Positive
JO9	28	M	Positive
JO10	24	M	Positive
AM01	74	M	Positive
AM02	80	F	Positive
AM03	71	F	Positive
AM04	85	F	Positive
AM05	64	F	Positive
AM06	60	F	Positive
AM07	62	M	Positive
AM08	61	M	Positive
AM09	61	M	Positive
AM10	61	M	Positive

### 2.2 Determination of donor CMV status

Detection of antibodies against CMV was performed using enzyme immunoassays (CMV IgG ELISA QuimioLab). The serum was stored at −20°C and analyzed by ELISA. Briefly, the assay was performed using a microtiter plate with the following steps: reagents were equilibrated to room temperature, and a 1:40 dilution of wash buffer was prepared. 100 μL of sample diluent was added to the wells (except for blank, positive, and negative controls), followed by 10 µL of the specimen and controls as appropriate. The wells were sealed, mixed, incubated at 37°C for 20 min, then washed five times. Subsequently, 50 µL of HRP conjugate was added, and the plate was incubated again at 37°C for 20 min, followed by another washing step. Substrates A and B were added (50 µL and 30 μL, respectively), incubated for 10 min, and then added a stop solution (50 µL). The results were read using a microplate reader. No molecular testing was performed.

### 2.3 Flow cytometry

The PBMC of each study participant were thawed and washed in AIM-V medium (Gibco, ThermoFisher) to be used in all experiments. (i) Monocyte and natural killer subpopulations were evaluated in *ex vivo*. A volume of 1 × 10^6^ cells in 50 ul of PBS labeled with one of the following monoclonal antibody panels from Biolegend: *Monocytes*: with FITC anti-CD14 (M5E2), PE/Cy5 anti-CD16 (3G8), PE/Dazzle594 anti-HLA-DR. *Natural killer*: Pacific blueTM anti-CD3, FITC anti-CD56 (5.1H11), APC anti-CD57 (HNK-1), APC/FireTM750 anti-KLRG1(SA231A2), PECy7 anti-NKp30 (P30-15), PE anti -NKG2D (1D11). (ii) One million PBMCs were seeded in 96-well flat-bottom dishes for 72 h with a mixture of beads coupled to antibodies against CD3, CD28, and CD2 (Miltenyi Biotec) in a 2:1 ratio (PBMCs: beads) grown in AIM-V medium (Thermo Fisher Scientific), This stimulation ensures sufficient activation and upregulation of key markers, such as PD1, which reflect the activation status and differentiation potential of the T cells (Cossarizza et al., 2022). After incubation, the cells were washed with PBS and labeled with of the following monoclonal antibody panels from Biolegend: Pacific blue™ anti-CD3, Brillant violet 510™ anti-CD4^+^ (SK3), PE/Dazzle594 anti-CD8 (SK1), FITC anti-CD45RO (UCHL1), Alexafluor700 anti-CD62L (DREG-5b), APC anti-CD57 (HNK-1) y APC/FireTM750 anti-KLRG1(SA231A2). It was incubated for 20 min at 4°C. All anti-human antibodies were used at the concentrations recommended by the manufacturer. Flow cytometry data were acquired using FACS Aria IIIu (BD) and analyzed using FlowJo Software V10 (BD).

### 2.4 FlowSOM multidimensional analysis

FlowSOM (SOM: self-organizing map) is an automated, unsupervised analysis tool that combines clustering and dimensionality reduction to simplify the complex distribution of labeled cells in a sample. This algorithm transforms data into two-dimensional graphs, where the distribution of events reflects the organization of cell populations co-expressing the evaluated markers (Van Gassen et al., 2015). The algorithm comprises four main steps: (i) reading the data, (ii) constructing a self-organizing map (SOM), (iii) building a minimum spanning tree, and (iv) performing meta-clustering to facilitate result interpretation. The relative percentage of a cell population is calculated as the proportion of events (cells) assigned to a cluster relative to the total number of events in the dataset. The data was analyzed following the guide of the Flowsom plugin for FlowJo (FlowJo, 2024).

### 2.5 Citrus analysis

Citrus (Cluster Identification, Characterization, and Regression) is an unsupervised analysis tool designed for identifying and stratifying cell subpopulations in multicolor flow cytometry datasets. It applies hierarchical clustering to identify cell clusters within the dataset, calculates descriptive features for each cluster, and employs regularized machine learning methods to determine the most representative cell subsets for each condition (Bruggner et al., 2014). For this study, exported FCS files were pre-processed by gating single cells and lymphoid regions based on FSC-A vs. FSC-H and FSC-A vs. SSC-A, respectively. Data transformation was performed using the ArcSinh function with a co-factor of 150 for conventional flow cytometry to normalize the data distribution. Each FCS file was downsampled to 10,000 events and pooled for hierarchical clustering based on the expression of nine cell markers. The Citrus package (v2.7) in R was used to identify differentially expressed clusters between experimental groups. Only clusters containing >2,500 cells were considered for analysis. Two algorithms, SAM (Significance Analysis of Microarrays) and PAMR (Prediction Analysis for Microarrays), were employed. SAM assessed the false discovery rate (FDR) with a p-value threshold of 1%, while PAMR identified the minimum and total number of clusters distinguishing between groups. Clusters with an error rate ≤10% and FDR = 0 were further analyzed using a Mann-Whitney test in Prism V9 to evaluate the statistical significance of differences in cluster abundances (Lalinde-Ruiz et al., 2021).

### 2.6 Statistical analysis

Statistical analyzes were performed on Prism V9 software (GraphPad). To compare groups (Young vs. Older), we applied non-parametric tests, Mann-Whitney and Kruskal–Wallis tests. For statistical differences p values <0.05 were considered statistically significant.

## 3 Results

### 3.1 Older adults exhibit an increase in intermediate and non-classical monocytes

**FIGURE 1:**
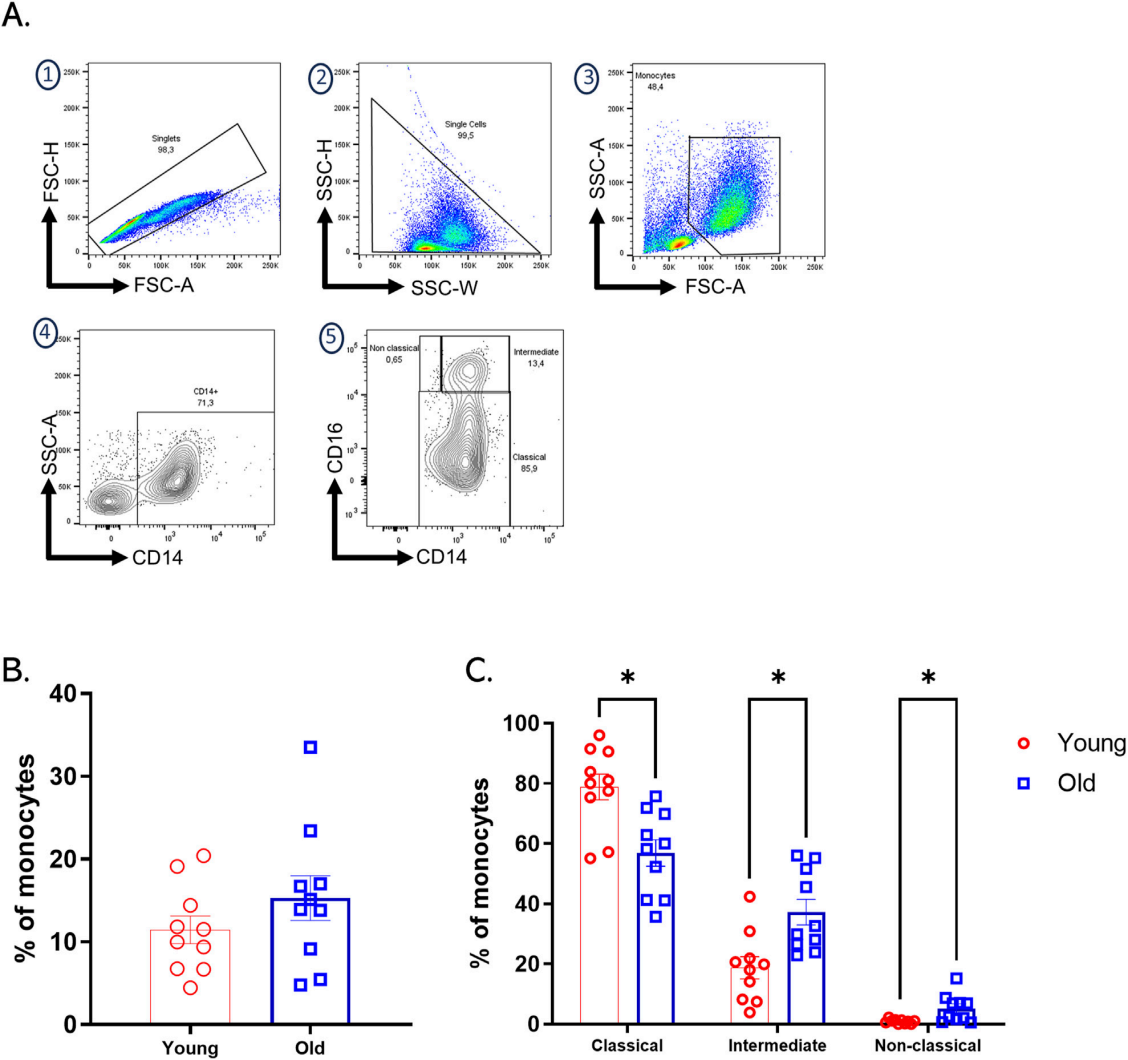
Increased intermediate and non-classical monocytes subpopulation in old adults. **(A)** The gating strategy for identifying monocyte subpopulations in PBMCs involved initial selection based on FSC-H vs. FSC-A parameters, followed by SSC-H vs. SSC-W analysis to isolate single cells. Monocyte selection was performed using FSC-A vs. SSC-A to differentiate cells by size and complexity. Monocyte subpopulations were then identified based on the differential expression of CD14 and CD16: classical monocytes (CD14^++^CD16^−^), intermediate monocytes (CD14^+^CD16^+^), and non-classical monocytes (CD14^dim^CD16^+^). **(B)** Bar plots of percentages of total monocytes of each group of age. **(C)** Bar graph showing the percentage of classical, intermediate, and non-classical monocyte subpopulations assessed by flow cytometry on expression differential CD14 and CD16 on PBMCs from twenty healthy donors, young (red bars), and older (blue bars) groups. A nonparametric t-test was performed with unpaired Mann-Whitney test data to compare young and old group. Data presented as means ± SEM (**p < 0.01; *p < 0.05).

Monocytes play a fundamental role in the initiation and resolution of inflammatory processes. Based on the differential expression of CD14 and CD16, flow cytometry analysis of monocytes allows the definition of three subpopulations of monocytes: classic (∼85%), intermediate (∼5%), and non-classical (∼10%). The proportions of these subpopulations may change with age or in chronic diseases. For example, intermediate monocytes increase in chronic inflammation and cardiovascular diseases (Marimuthu et al., 2018). When analyzing blood samples from our twenty CMV + subjects and comparing them by age group, we found no significant differences between the total percentage of circulating monocytes (Figure 1B). However, when comparing monocyte subpopulations classified by differential expression of CD14 and CD16, we found a statistically significant increase in intermediate and non-classical monocytes in older adults (Figure 1C).

### 3.2 Increase in circulating natural killer (NK) cells in older adults

**FIGURE 2:**
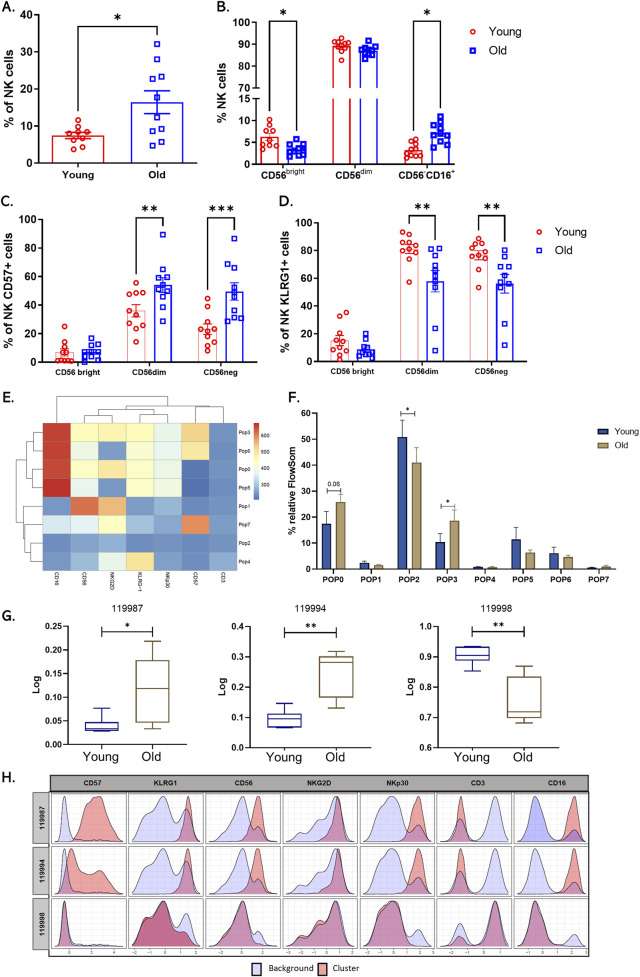
Increase in circulating natural killer cells and changes in NK cell receptor expression in older adults. **(A)** Bar graph shows the percentage of total circulating NK cells from twenty healthy donors, young (red bars), and older (blue bars) groups. **(B)** Bar graph showing the percentage of the three main subpopulations across a differential expression of CD56 y CD16. **(C)** Bar graph showing the percentage of NK cells expressing CD57. And **(D)** KLRG1. **(E)** Heat map of each marker in the eight populations determined by FlowSOM. **(F)** Bar graph of relative cell frequency of eight populations determined by FlowSOM between young adults (blue bars) and old adults (brown bars). **(G)** Boxplots showing the percentage of Relative abundance (Log10) of cluster 119987, 119994, and 119998 in the two groups, young adults (blue) and old adults (brown) (n = 10/group). **(H)** Histograms of CITRUS depicting the phenotype of cluster 119987, 119994, and 119998 (red histograms) relative to background expression (blue histograms) for each marker (CD57, KLRG1, CD56, NKG2D, NKp30, CD3, and CD16. A nonparametric t-test was performed with unpaired Mann-Whitney test data to compare young and old group. Data presented as means ± SEM (**p < 0.01; *p < 0.05).

NKs are cells of the innate immune system responsible for eliminating infected, malignant, or senescent cells; they represent about 5%–20% of total peripheral lymphocytes (Witkowski et al., 2020). In humans, NK subpopulations are classified according to the level of expression of the cytotoxicity marker CD56 (Tarazona et al., 2015). Mainly two subpopulations of NKs have been described; the first, comprising 90% of circulating NKs, are CD56dimCD16+ and highly cytotoxic, and the second are CD56bright CD16^+^ producers of cytokines such as IFNγ and TNFα (Witkowski et al., 2020). An additional population has been reported that is CD56^−^ CD16^+^, which is mainly found in patients with latent virus infections (Müller-Durovic et al., 2019). Aging affects the number, subpopulation distribution, and function of NKs. In line with the above, changes in these cells in our sample of volunteers were evaluated through a multicolor panel. The strategy for selecting NK cells and their subpopulations based on differential expression of CD56 and CD16 is outlined in Supplementary Figure 1A. Analysis of the data revealed that older adults have a significantly higher number of circulating NK cells in peripheral blood compared to younger individuals (Figure 2A). Additionally, a decrease in the CD56 bright subpopulation and an expansion of CD56^−^CD16^+^ cells were observed (Figure 2B).

### 3.3 Age-related changes in natural killer cell receptor expression

A balance between activating receptors (NKp30, NKp46, NKG2D) and inhibitory receptors (KIR, KLRG1) regulates natural killer cell activity. Aging and chronic viral infections, such as CMV, are associated with decreased expression of natural killer receptors and increased expression of inhibitory receptors and CD57 (Phan et al., 2017). CMV infection explicitly drives the expansion of mature and dysfunctional CD56^dim^CD16^+^ NK cell subsets expressing CD94, NKG2C, and inhibitory receptors (Reed et al., 2019). Furthermore, high KLRG1 expression in individuals older than 70 has been described to downregulate NK cell function by activating the metabolic sensor AMPK (Müller-Durovic et al., 2016). Despite the existing body of research, discrepancies persist regarding the behavior of NK receptors with age. In this context, we measured the expression of NKG2D, NKp30, KLRG1, and CD57 in NK subpopulations in the twenty CMV+ donors and compared them by age group. We found increased CD57 expression and decreased KLRG1 expression, mainly in CD56dim and CD56neg subpopulations (Figures 2C, D). NKG2D and NKp30 showed no significant differences between the groups with manual analysis (Supplementary Figure 1B).

We then performed an unsupervised automated analysis using the FlowSOM algorithm on the NK cell samples to identify eight clusters with differential expression of the markers from the panel used. When comparing these subpopulations, we observed that populations 0 (CD3^−^CD56dimCD16^++^CD57^−^KLRG1^+^NKG2D^+^NKp30^Low^) and 3 (CD3^−^CD56dimCD16^++^CD57^+^KLRG1^+^NKG2D^+^NKp30^Low^), which represent subpopulations of NK cells mature and cytotoxic, showed a significant increase in their presence in older adults. We found a cluster two characterized by being negative for all the markers included in the panel, and this cluster is increased in young individuals (Figures 2E, F).

Furthermore, to corroborate our findings, we performed automated analysis using the Citrus algorithm, which allowed us to identify three NK cell clusters with statistical differences between older and younger adults. Clusters 119987 and 119994 presented an expression profile like that of populations 0 and 3 established by FlowSOM and are increased in older adults (Figure 2F). On the other hand, the cluster 119998, characterized by an absence of expression of all markers, showed a significant decrease in older adults compared to young adults (Figures 2G, H).

### 3.4 Analysis of T cell subpopulations with aging: findings from FlowSOM and citrus

**FIGURE 3:**
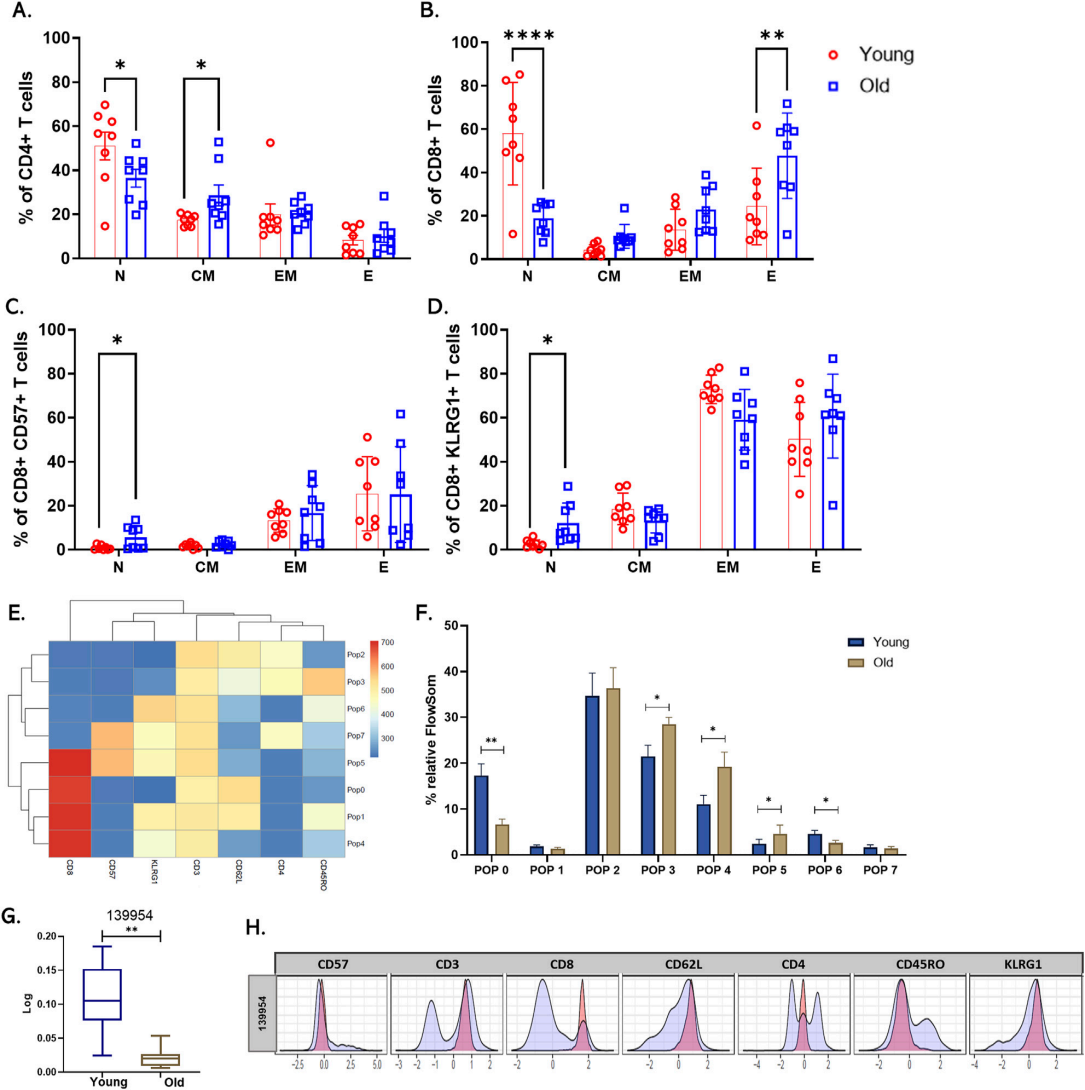
Changes of T cell subpopulations with aging. **(A)** Scatter point bars showing the percentage of each memory subsets: CD62L + CD45RO− naïve T cells (N cells), CD62L + CD45RO + central memory T cells (CM cells), CD62L−CD45RO + effector memory T cells (EM cells) and CD62L−CD45RO− terminal effector T cells (E cells) within CD3^+^CD4^+^. **(B)** CD3^+^CD8^+^ compartments. Young (red bar) and Old (blue bar) groups. **(C, D)** The Senescence markers expression on CD4^+^ and CD8^+^ T cells was assessed by flow cytometry on PBMCs from ten healthy donors within memory subsets of CD4^+^ and CD8^+^ T cells. **(E)** Heat map of each marker in the eight populations determined by FlowSOM. **(F)** Bar graph of relative cell frequency of eight populations determined by FlowSOM between young adults (blue bars) and old adults (brown bars). **(G)** Boxplots showing the percentage of Relative abundance (Log10) of cluster 139954 in the two groups, young adults (blue) and old adults (brown) (n = 10/group). **(H)** Histograms of CITRUS depicting the phenotype of cluster 139954 (red histograms) relative to background expression (blue histograms) for each marker (CD57, CD3, CD8, CD62L, CD4, CD45RO, and KLRG1). A nonparametric t-test was performed with unpaired Mann-Whitney test data to compare young and old group. Data presented as means ± SEM (**p < 0.01; *p < 0.05).

Thymic involution experienced throughout life leads to a reduction in T cells turnover (Zaoqu et al., 2023). Consequently, aging is characterized by a marked decrease in the pool of naïve T cells and a notable increase in highly differentiated T cell populations, which display senescent characteristics. The senescent phenotype of T cells is manifested by an increase in the expression of maturation receptors on the cell surface, such as CD57 and KLRG1 (Akbar et al., 2016; Henson et al., 2015), and a decrease in the responsiveness of the T cell receptor (TCR) to antigens (Pereira et al., 2020). In this context, we evaluated the distribution of memory populations in CD4^+^ and CD8^+^ compartments in the twenty CMV + donors and compared between age groups. To assess memory subpopulations, we used the differential expression of CD62L and CD45RO membrane receptors to identify the following T cell subsets: (a) CD62L + CD45RO− naive T cells (N cells), (b) CD62L + CD45RO + central memory T cells (CM cells), (c) CD62L−CD45RO+ effector memory T cells (EM cells), and (d)CD62L−CD45RO− terminal effector memory T cells (E cells), (Supplementary Figure 1C). We found that naive CD4^+^ T cells (N) decreased, and the central memory (CM) subpopulation increased (Figure 3A). We evaluated the differential expression of senescence markers, such as CD57 and KLRG1, between T cells from old and young adults. We observed a significant increase in the expression of these markers in the naïve CD8^+^ T cell subpopulation in older adults (Figures 3C, D). In contrast, we found no significant changes in the expression of these markers in CD4^+^ T cell subpopulations between young and old adults (data not shown).

An automated analysis using FlowSOM was performed to validate the manual analysis results, which showed four increased clusters in older adults. (i) The cluster 3 (Pop3: CD3^+^CD4^+^CD45RO^+^CD62^low^), characterized as a CD4^+^ effector memory T cell population that does not show markers of senescence; (ii) The cluster 4 (Pop4: CD3^+^CD8^+^KLRG1^+^), identified as a population of terminally differentiated effector CD8^+^ T cells expressing KLRG1; (iii) The cluster 5 (Pop5: CD3^+^CD8^+^KLRG1^+^CD57^+^), described as a terminally differentiated population that expresses both senescence markers; and (iv) cluster 6 (Pop6: CD3^+^KLRG1^+^ negative for CD4 and CD8), possibly corresponding to an NKT cell population excluded in the manual analysis (Figures 3E, F). In contrast to the increase in these four cell clusters in older adults, a cluster of CD8^+^ T cells (pop0 CD3^+^CD8^+^CD62L^+^) was detected increase in young individuals. This increase in the naïve CD8 population detected by FlowSOM in young people coincides with the significant increase observed in the group of young individuals in the manual analysis (see Figure 3B). In contrast to the FlowSOM findings, Citrus identified one cluster that significantly increased in young individuals (Figures 3G, H) and shares characteristics with cluster 0 (pop0 CD3^+^CD8^+^CD62L^+^) identified in FlowSOM. These results suggest that the most notable change in T cells associated with aging is the decreased naïve CD8^+^ T cell subpopulation.

## 4 Discussion

Monocytes play a fundamental role in the immune response due to their phagocytic capacity, which is necessary for the processing and presentation of antigens and the production of cytokines. In aging, monocytes are critical cells in age-related immune dysfunction (Yang et al., 2014). Our study observed a decrease in classical CD14++CD16^−^ monocytes and an increase in CD14^+^CD16^+^ intermediate monocytes in older adults. The intermediate monocytes are characterized as proinflammatory cells that produce cytokines such as TNFα and IL-6. These cytokines have been associated with chronic low-grade inflammation or *inflammaging* (Pence, 2019). Furthermore, previous studies have shown an association between variation in circulating monocyte subpopulations and the development of diseases such as coronary heart disease (Ketelhuth et al., 2019) and various types of cancer (Pence, 2019).

When we analyzed NKs in our sample, we observed a significant increase in CD56neg cells, increased expression of CD57, and a notable decrease in CD56bright cells in older adults. These findings align with previous immunosenescence research reporting a decrease in immature NK and an increase in CD56dim cells with CD57 expression (Tarazona et al., 2015; Müller-Durovic et al., 2019; Müller-Durovic et al., 2016; Campos et al., 2014). Studies have shown that CD56neg cells are less functional regarding cytotoxicity and responsiveness, especially in CMV + individuals (Müller-Durovic et al., 2019). When evaluating the expression of receptors such as NKG2D, NKp30, CD57, and KLRG1, a decrease in the expression of KLRG1 was found in CD56dim cells from older adults. Elevated expression of KLRG1 has been correlated with a reduction in the proliferative capacity and effector function of NKs (Müller-Durovic et al., 2016; Monsiváis-Urenda et al., 2010). However, the literature needs to be more conclusive regarding the expression of KIR receptors, and additional studies considering different age groups and CMV infection status are required to understand their influence in our population better. The expansion of two mature NK populations, CD56dimCD16+ CD57^+^, and CD56dimCD16+ CD57^−^, was identified, which may be related to the adaptive response during chronic viral infections such as CMV (Witkowski et al., 2020; Bigley et al., 2016). It is crucial to underscore that CMV infection is a significant factor in studies of immune correlation and aging, and more research needs to be conducted with a larger sample to differentiate its effect from physiological aging on the immune system.

In this study, using the differential expression of CD62L and CD45RO, the distribution of memory subpopulations in the T cells was determined. A significant decrease in the naïve cell subpopulation was observed in CD4^+^ and CD8^+^ t cells in the older adult group. Although the total number of T cells remains relatively constant with aging, reducing the naïve T cells is a hallmark of immunosenescence (Pawelec, 2018). This decline in naïve T cells could explain, at least in part, the increased susceptibility of older adults to infectious diseases, chronic inflammatory diseases, and cancer (Zhou and McElhaney, 2011; Goronzy et al., 1950). Furthermore, we observed a significant increase in terminally differentiated effector CD8^+^ T cells in older adults. This increase in T cell effectors has been observed in both aging and chronic infections, such as those caused by CMV, which is the case for our individuals (Elwenspoek et al., 2017). In our work, we found a significant increase in the expression of CD57 and KLRG1 with age, especially in the effector and effector memory subpopulations. These results align with what has been reported in the literature, where an increase in the CD57+KLRG1+ T cells has been observed in older adults (Henson et al., 2015; Onyema et al., 2015; Xu et al., 2019; Dolfi et al., 2013; Onyema et al., 2012; Ouyang et al., 2003).

In conclusion, our analysis suggests potential alterations in the distribution and expression of senescence markers of immune cell subpopulations, which may be influenced by factors such as CMV infection. Specifically, we observed an increase in pro-inflammatory intermediate monocytes and NK cells CD56neg, a decrease in naïve T cells, and an increased expression of senescence markers in effector CD8^+^ T cells. However, as no comparison group of CMV-seronegative individuals was included, these findings should be interpreted cautiously. Further studies with defined CMV-seropositive and seronegative groups are necessary to confirm the specific impact of CMV on immunosenescence in Colombia’s population, which has a high incidence of CMV infection.

## Data Availability

The raw data supporting the conclusions of this article will be made available by the authors, without undue reservation.

## Supplementary material

SUPPLEMENTARY FIGURE S1 Gating analysis. **(A)** Analysis strategy for identifying NK cells and CD56bright, CD56dim, and CD56neg subpopulations. **(B)** Bar graph showing the percentage of NK cells expressing NKG2D and NKp30. **(C)** Analysis strategy. In T cells, the T cells population was defined based on its size and complexity characteristics using the parameters (FSC-A and SSC-A), the CD3+ cell region was delimited, and within these, the CD4 and CD8 populations followed by the analysis of CD62L and CD45RO expression for the memory populations N (Naïve: CD62L^+^, CD45RO^−^), CM (Central memory: CD62L^+^, CD45RO^+^), EM (memory effectors: CD62L^−^, CD45RO^+^) and E (Effectors: CD62L^−^, CD45RO^-^).
