# Supplementary figures and images for: Markers of immunosenescence in CMV seropositive healthy elderly adults
Source: Front Aging. 2025 Jan 23;5:1436346. doi: 10.3389/fragi.2024.1436346 (PMC11798936; doi:10.3389/fragi.2024.1436346)

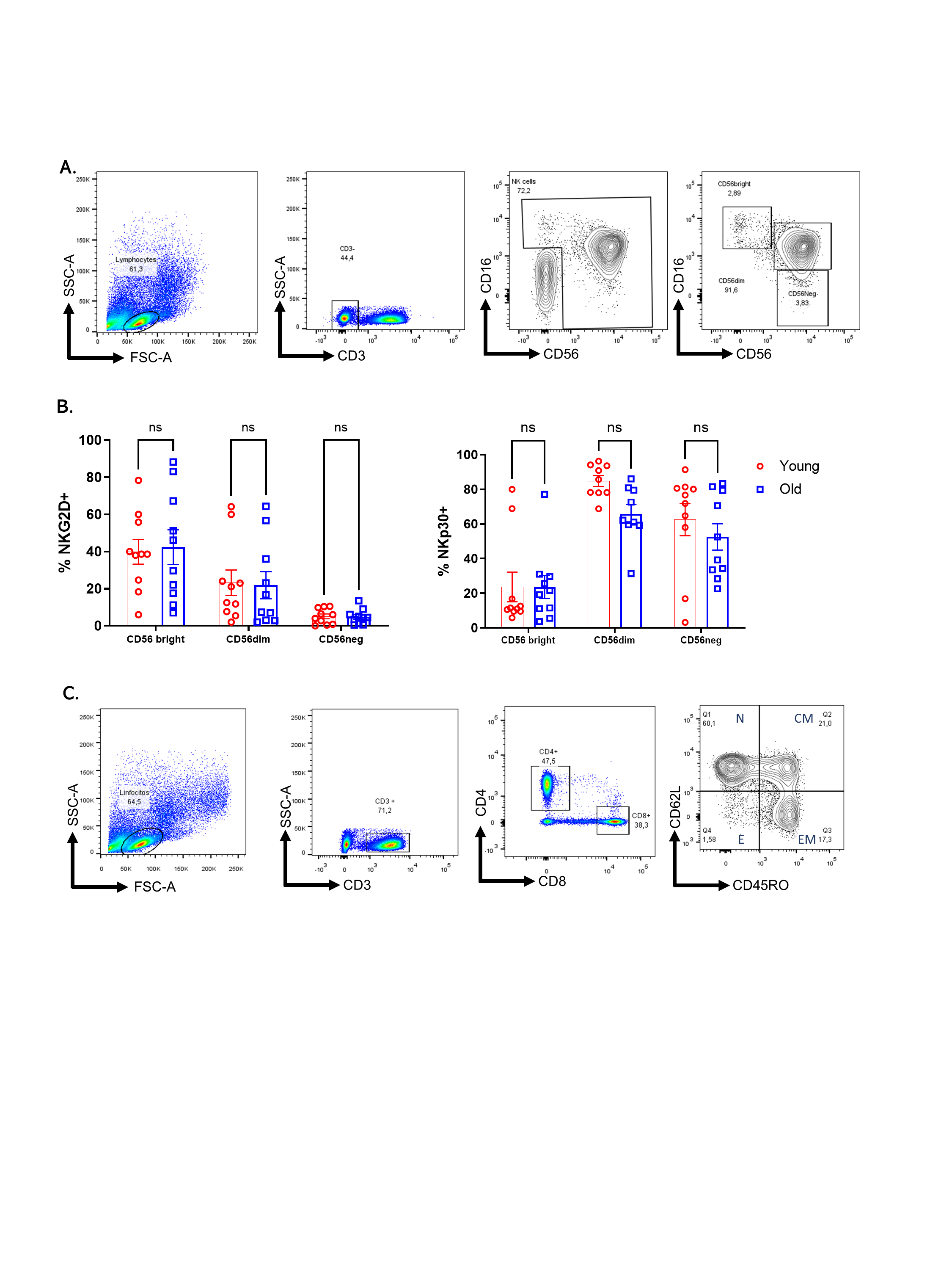

Supplement: Supplementary file 1 [file Image1.tif]
